# Supplementary material for: From sequence to enzyme mechanism using multi-label machine learning
Source: BMC Bioinformatics. 2014 May 19;15:150. doi: 10.1186/1471-2105-15-150 (PMC4229970; doi:10.1186/1471-2105-15-150)
Supplement: Additional file 2 — Java code of ml2db. Additional file ml2db_code.tar.gz contains the Java source code to run the multi-label machine learning experiments and save the results to database. The code’s Javadoc is included. [file 1471-2105-15-150-S2.zip › additional file 2/ml2db/ml2db/doc/mulan/data/class-use/InvalidDataFormatException.html]

Uses of Class mulan.data.InvalidDataFormatException


JavaScript is disabled on your browser.


- Overview
- Package
- Class
- Use
- Tree
- Deprecated
- Index
- Help

- Prev
- Next

- Frames
- No Frames

- All Classes

## Uses of Class mulan.data.InvalidDataFormatException

- Packages that use InvalidDataFormatException

  | Package | Description |
  | --- | --- |
  |  |  |
  | --- | --- |
  | mulan.classifier.meta |  |
  | mulan.data |  |
- - ### Uses of InvalidDataFormatException in mulan.classifier.meta

    Methods in mulan.classifier.meta that throw InvalidDataFormatException

    | Modifier and Type | Method and Description |
    | --- | --- |
    |  |  |
    | --- | --- |
    | `static MultiLabelInstances` | HierarchyBuilder.`createHierarchicalDataset(MultiLabelInstances mlData, LabelsMetaData metaData)` Creates the hierarchical dataset according to the original multilabel instances object and the constructed label hierarchy |
  - ### Uses of InvalidDataFormatException in mulan.data

    Methods in mulan.data that throw InvalidDataFormatException

    | Modifier and Type | Method and Description |
    | --- | --- |
    |  |  |
    | --- | --- |
    | `MultiLabelInstances` | MultiLabelInstances.`reintegrateModifiedDataSet(weka.core.Instances modifiedDataSet)` If `Instances` data set are retrieved from `MultiLabelInstances` and post-processed, modified by custom code, it can be again reintegrated into `MultiLabelInstances` if needed. |

    Constructors in mulan.data that throw InvalidDataFormatException

    Constructor and Description

    `MultiLabelInstances(java.io.InputStream arffDataStream,
    java.io.InputStream xmlLabelsDefStream)`

    Creates a new instance of `MultiLabelInstances` data from the supplied `InputStream`
    data source.

    `MultiLabelInstances(java.io.InputStream arffDataStream,
    int numLabelAttributes)`

    Creates a new instance of `MultiLabelInstances` data from the supplied `InputStream`
    data source.

    `MultiLabelInstances(weka.core.Instances dataSet,
    LabelsMetaData labelsMetaData)`

    Creates a new instance of `MultiLabelInstances` data from existing `Instances`
    and `LabelsMetaData`.

    `MultiLabelInstances(weka.core.Instances data,
    java.lang.String xmlLabelsDefFilePath)`

    Creates a new instance of `MultiLabelInstances` data.

    `MultiLabelInstances(java.lang.String arffFilePath,
    int numLabelAttributes)`

    Creates a new instance of `MultiLabelInstances` data.

    `MultiLabelInstances(java.lang.String arffFilePath,
    java.lang.String xmlLabelsDefFilePath)`

    Creates a new instance of `MultiLabelInstances` data.

- Overview
- Package
- Class
- Use
- Tree
- Deprecated
- Index
- Help

- Prev
- Next

- Frames
- No Frames

- All Classes
